# Supplementary material for: Effect of Continuous Positive Airway Pressure on Changes of Plasma/Serum Ghrelin and Evaluation of These Changes between Adults with Obstructive Sleep Apnea and Controls: A Meta-Analysis
Source: Life (Basel). 2023 Jan 4;13(1):149. doi: 10.3390/life13010149 (PMC9865449; doi:10.3390/life13010149)
Supplement: Supplementary file 1 [file life-13-00149-s001.zip › life-2067014-supplementary.pdf]

**I. The Joanna Briggs Institute (JBI) Critical Appraisal Checklist for case-control study (last amended in 2017)**<br>**Website:** https://joannabriggs.org/critical_appraisal_tools<br>https://wiki.joannabriggs.org/display/MANUAL/Appendix+7.2+Critical+appraisal+checklist+for+case-control+studies				
Major Components	Response options			
1. Were the groups comparable other than the presence of disease in cases or the absence of disease in controls?	Yes	No	Unclear	Not applicable
2. Were cases and controls matched appropriately?	Yes	No	Unclear	Not applicable
3. Were the same criteria used for identification of cases and controls?	Yes	No	Unclear	Not applicable
4. Was exposure measured in a standard, valid and reliable way?	Yes	No	Unclear	Not applicable
5. Was exposure measured in the same way for cases and controls?	Yes	No	Unclear	Not applicable
6. Were confounding factors identified?	Yes	No	Unclear	Not applicable
7. Were strategies to deal with confounding factors stated?	Yes	No	Unclear	Not applicable
8. Were outcomes assessed in a standard, valid and reliable way for cases and controls?	Yes	No	Unclear	Not applicable
9. Was the exposure period of interest long enough to be meaningful?	Yes	No	Unclear	Not applicable
10. Was appropriate statistical analysis used?	Yes	No	Unclear	Not applicable

**Q. The National Institutes of Health (NIH) quality assessment tool for before-after (Pre-Post) study with no control group**<br>**Website:** https://www.nhlbi.nih.gov/health-topics/study-quality-assessment-tools			
Major Components	Response options		
1. Was the study question or objective clearly stated?	Yes	No	Cannot Determine/ Not Applicable/ Not Reported
2. Were eligibility/selection criteria for the study population prespecified and clearly described?	Yes	No	Cannot Determine/ Not Applicable/ Not Reported
3. Were the participants in the study representative of those who would be eligible for the test/service/intervention in the general or clinical population of interest?	Yes	No	Cannot Determine/ Not Applicable/ Not Reported
4. Were all eligible participants that met the prespecified entry criteria enrolled?	Yes	No	Cannot Determine/ Not Applicable/ Not Reported
5. Was the sample size sufficiently large to provide confidence in the findings?	Yes	No	Cannot Determine/ Not Applicable/ Not Reported
6. Was the test/service/intervention clearly described and delivered consistently across the study population?	Yes	No	Cannot Determine/ Not Applicable/ Not Reported
7. Were the outcome measures prespecified, clearly defined, valid, reliable, and assessed consistently across all study participants?	Yes	No	Cannot Determine/ Not Applicable/ Not Reported
8. Were the people assessing the outcomes blinded to the participants' exposures/interventions?	Yes	No	Cannot Determine/ Not Applicable/ Not Reported
9. Was the loss to follow-up after baseline 20% or less? Were those lost to follow-up accounted for in the analysis?	Yes	No	Cannot Determine/ Not Applicable/ Not Reported
10. Did the statistical methods examine changes in outcome measures from before to after the intervention? Were statistical tests done that provided p values for the pre-to-post changes?	Yes	No	Cannot Determine/ Not Applicable/ Not Reported
11. Were outcome measures of interest taken multiple times before the intervention and multiple times after the intervention (i.e., did they use an interrupted time-series design)?	Yes	No	Cannot Determine/ Not Applicable/ Not Reported
12. If the intervention was conducted at a group level (e.g., a whole hospital, a community, etc.) did the statistical analysis take into account the use of individual-level data to determine effects at the group level?	Yes	No	Cannot Determine/ Not Applicable/ Not Reported
